# Rate of publication hastens, but number of publications slows academic promotion

**DOI:** 10.1371/journal.pone.0276616

**Published:** 2022-10-26

**Authors:** Jackson B. Pickett, Paul Savala

**Affiliations:** 1 Office of Sponsored Programs, Saint Edwards University, Austin, Texas, United States of America; 2 Department of Mathematics, Saint Edwards University, Austin, Texas, United States of America; Sapienza University of Rome, ITALY

## Abstract

Openings for an assistant professor often attract a hundred or more applicants. This allows hiring committees to select highly productive candidates based on their number of publications. Applicants with more rapid publication would be hired with little or no postgraduate experience, but those with slower rates of publication would need more postgraduate experience. Our results show an association of more postgraduate experience, slower rates of publication, a smaller research group, and slower promotion when years are measured from PhD granting; conversely little or no postgraduate experience is generally associated with more rapid publication, a larger research group, and faster promotion. These results suggest the unexpected result that the number and rate of publication have opposite effects on the years from PhD granting to promotion which parametric survival analysis using a log-logistic distribution with gamma frailty confirmed. Statistical analysis revealed that number and rate of publication are reciprocal suppressor variables which were individually weaker predictors of years to promotion, but much more powerful when combined. Intuitively, this is probably because number and rate of publication contain information about other variables with: (1) number of publications being associated with more postgraduate experience, a smaller research group, and slower rates of publication; and (2) rate of publication being associated with a larger research group, and less postgraduate experience. Further, we found that promotion committees closely follow institutional tenure policy requiring promotion a fixed number of years after hiring as an assistant professor which may partially explain why promotion committees fail adjust the number and rate of publication for research group size as fairness in promotion might favor. Our results suggest that both postgraduate experience and research group size influence a professor’s career.

## Introduction

A professor’s career typically starts with the granting of an advanced degree, often a PhD, and can be followed by years of postgraduate experience before being hired as an assistant professor. For many years, the supply of PhDs has greatly exceeded the number of assistant professor positions leading to more than one hundred applicants for an advertised position [1–3]. For those not hired immediately after completing their PhD, many become postdocs to increase their number of publications hoping to increase their chance of becoming an assistant professor [2, 4–10].

Many factors beyond postgraduate experience influence hiring and promotion in an academic setting. In addition to postdoctoral experience [9, 11–17], working as part of a research group would be expected to influence hiring and promotion. Large research groups are typical of some fields, such as physics, and have become increasingly important in the social sciences including geography [12, 17–20]. In the geosciences over about 40 years, from the late 1950s to the late 1990s, research team size has doubled from 1.50 to 3.13 [17]. A problem associated with larger research teams, typically estimated by number of authors per paper, is how to fairly allocate credit for collective productivity. When considered for tenure after serving a probationary period of five to seven years [6, 7, 21–27], should their number and rate of publication be corrected for research group size, perhaps by dividing by the number (N) of authors or something less such as the square root of N [12, 18, 20, 24, 28, 29]? Since empirical studies show that productivity is related to research group size, it is plausible that promotion committees would adjust the number and rate of publication for research group size so professors would not gain credit for more than their 1/N share of the group’s productivity [12, 18, 20, 24, 28]. Alternatively, the tenure clock might constrain the time to promotion to an interval of five to seven years after hiring, offering little opportunity for more rapid promotion [21, 25, 26].

### Review of the literature

In the late 1960s and early 1970s Civil Rights legislation was passed banning discrimination based on sex [30]. This legislation led to studies investigating the possibility of discrimination in the hiring and promotion of women [23, 25, 27, 30–32]. It was recognized that hiring and promotion could be influenced by a variety of variables including demographic traits, field of specialization, work environment, and nature of employing institution [23, 25, 27, 30–32]. To detect an effect of gender it would be necessary to correct for the influence of these variables which was done with linear and logistic regression, and survival analysis [23, 25, 27, 30–32]. Ceci and colleagues [33] have reviewed studies of the nexus of gender, academic productivity, and time to promotion and rated the evidence that gender influences promotion as “thin” [33]. However, these regression studies have identified other consistently significant variables including that: (1) number of publications influences time to promotion; (2) the years to gain tenure, which is usually coincidental with promotion to become an associate professor, are dictated by institutional tenure policy forcing promotion about five to seven years after hiring as an assistant professor; (3) increasing years between PhD granting and hiring slow promotion; and (4) the number of moves to another institution an assistant professor slows promotion to become an associate professor [4, 6, 7, 23, 25, 29, 31, 32, 34–38]. The rate of publication should hasten promotion but has rarely been evaluated by regression studies [37]. It is likely that increasing grant funding will encourage research collaboration, increasing research group size and productivity [4, 6, 9, 10, 28, 35, 38]. Similarly, affiliation with a top ranked department should favor collaboration and rate of publication [11, 13, 39]. It is likely that some of these variables will complement each other.

One pair of variables that should complement each other is the number and rate of publication. The literature suggests that actual hiring and promotion are based on the number of publications [23, 25, 27, 30–32]. If years to promotion included postgraduate experience, then the number of publications needed for hiring as an assistant professor would require: (1) less postgraduate experience for candidates working in larger research groups who would have a more rapid rate of publication [12, 18, 20, 24, 28]; and (2) more postgraduate experience for applicants who work in smaller research groups who have a slower rate of publication [12, 18, 20, 24, 28, 29]. As a result, the effect of postgraduate experience and research group size would tend to cause number and rate of publication to have opposite effects on cumulative years from PhD granting to promotion. We will present evidence to support this unexpected, and possibly novel, result. Further, we have discovered that the number and rate of publication are reciprocal suppressor variables [40–46]. Suppressor variables are individually relatively weak predictors, but when combined gain the ability to explain much more of the variance than either variable individually; this is true of the number and rate of publication [40–46]. A last factor influencing promotion is how tenure and promotion committees influence the timing of promotion [4, 7, 21, 23, 26, 29, 33].

Institutional tenure policy requires that promotion decisions be made within a fixed time frame, typically from five to seven years; this will tend to cause the years to promotion to be approximately constant over many years [4, 7, 21, 23, 26, 29, 47]. However, in making tenure and promotion decisions, committees are expected to adjust the number and rate of publication by dividing by research group size, N, or as Bikard, Murray, and Gans suggest for allocating credit for citations, by approximately the square root of N, or N^½^. Allocation for the contribution of authors to the publications in a field may follow Price’s square root law “…that half of the literature on a subject will be contributed by the square root of the total number of authors publishing in that area” [48, 49]. If this is true, then a correction might be needed: (1) so that professors working in larger research groups can be more fairly be compared to those working in smaller research groups; and (2) to compensate for the diminishing returns of working in larger groups or the fraction of time a professor spends doing research (17–19). We will present evidence that promotion committees do adjust both number and rate of publication for research group size but unexpectedly the effect of this correction is slight.

### Hypotheses to be tested

Hypothesis 1. The number and rate of publications will have opposite effects on the timing of promotion with number slowing promotion and rate hastening promotion.
Hypothesis 2. Increasing research group size will be associated with an increase in number and rate of publication.
Hypothesis 3. The relationship between cumulative years until promoted counted from PhD granting vs. years until hired as an assistant professor will be moderated by the rate of publication.
Hypothesis 4. Increasing postgraduate experience will increase the number of publications when a professor is hired and promoted, but the rate of publication will be independent of postgraduate experience.
Hypothesis 5. Professors working in larger research groups, of two or more, and those with more postgraduate experience, a year or more, will be hired and promoted with about the same number of cumulative publications. Likewise, the incremental years to promotion will be about the same in both groups.
Hypothesis 6. Tenure and promotion committees will appear to divide measures of productivity by research group size, N, or fractional powers of N, N^k^, such as the square root of N, N^½^, when making tenure and promotion decisions.
Hypothesis 7. Institutional policy will force tenure decisions, and typically promotion to become an associate professor, to occur within about five to seven years from hiring as an assistant professor. There will tend to be less rigid, but similar expectation of the number of years until promotion to become a full professor.

## Methods

### Research design, variable definitions, and sources of data

This study identified professors hired by PhD granting departments of geography between 1991 and 2010. All information collected was from public sources. Professors of geography hired between 1991 and 2010 at PhD granting institutions were identified by being listed in the Association of American Geographers’ Guides and Directories and traced until January 2020. During this period 3,808 geography PhDs were granted and 935 assistant professors were hired by PhD-granting geography departments, or about 24.6% of geography PhDs granted. About 9.3% of professors had PhDs granted before 1991, usually first working in business or government. A total of 74 departments in 36 states were included.

Table 1 lists all the variables used in this study.

**Table 1:** Variable definitions.

Variable Name	Variable Definition
Publications or Articles Hired, Associate, Professor	Cumulative publications or articles when hired as assistant professor, or promoted to become an associate or full professor starting five years before PhD granted. Publications included articles, book reviews, proceedings papers, editorial material, and book chapters.
Publication or Article Rate Associate, Professor	Cumulative publications or articles divided by years from PhD granting until promoted to become an associate or full professor. Publications included articles, book reviews, proceedings papers, editorial material, and book chapters.
Citations as Associate, or Full Professor	Cumulative citations of papers published when promoted to become an associate or full professor
Number of Authors per Publication	Average number of authors per publication excluding publications with > 30 authors
Year of PhD	Year PhD was granted
Top PhD, Top Assistant or Associate	PhD, hiring, or promotion at Clark University, Ohio State University, Pennsylvania State University, State University of New York-Buffalo, Syracuse University, University of California-Berkeley, University of California-Los Angeles, University of California-Santa Barbara, University of Colorado-Boulder, University of Minnesota-Twin Cities, University of Washington, or University of Wisconsin-Madison.
Years Until Hired, Associate, Professor	Years between PhD granting and hiring as an assistant professor, or promotion to become an associate or full professor
Moves Number, to PhD- Granting Institution	Number of moves as assistant professor before promotion, or a move as an assistant professor to a PhD-granting institution
Region	States divided into five regions:
	Lakes: Hired in state of IL, IN, MI, OH, or WI
	New Engl: Hired in state of CT, DC, DE, MA, MD, ME, NH, NJ, NY, PA, RI, or VT
	Plains: Hired in state of CO, IA, ID, KS, MN, MO, MT, ND, NE, SD, UT, or WY
	South: Hired in state of AL, AR, AZ, FL, GA, KY, LA, MS, NC, NM, OK, SC, TN, TX, VA, or WV
	West: Hired in state of AK, CA, HI, NV, OR, or WA
Geography	Research focus within geography
Gender	Male or female

Publications, articles, and citations were those listed by Thomas Reuters’ Web of Science Core Collection. Publications included articles, book reviews, proceedings papers, editorial material, and book chapters. Cumulative publications included those published five years before a professor’s PhD was granted, but incremental publications were measured from hiring or most recent promotion. Cumulative years were counted from the year of PhD granting; incremental years were counted from year of PhD granting or most recent change in academic rank. The cumulative (or incremental) rate of productivity was calculated by dividing the cumulative (or incremental) publications or articles by cumulative (or incremental) years. The number of authors per paper excluded those with more than 30 authors. Year of granting of the professor’s PhD, hiring, and promotion were found in Association of American Geographers’ Guides and Directories, departmental websites, and university catalogs, when sources conflicted the year of PhD granting was verified using Proquest’s Dissertation and Theses database. Moves while an assistant professor were counted. Institutional tenure policy was determined by an internet survey in May of 2021. Professors promoted to become associate professors were within their tenure window if they were promoted within a year of institutional tenure policy.

### Descriptive statistics and regression models

Statistical analysis was done with Stata version 17. Descriptive statistics were calculated for groups of professors with variables compared by Student t-tests. Nested linear regression was used to evaluate whether the fit of quadratic regression was significantly better than that of linear regression. Moderation, or interaction of variables, was displayed with Stata’s margins and marginsplot commands [50]. Stata’s aic_selection_model with pe(0.8) and pe(0.05) used the Akaike information criteria (AIC) to select the best fitting Cox or parametric survival analysis regression model. When the AIC values of two similar regression models differed by no more than about two the simpler model was chosen.

### Survival analysis and frailty

Survival analysis models the time until an event, described by a survival function S(t), which is defined as the probability that an event occurs after time t. Survival functions can be parametric (given by a probability distribution) or semi-parametric, in which case a probability distribution is fit from the data, such as in the Cox proportional hazard model. When parametric models provide a good fit for the data, they are often preferable over semi-parametric models, as their parameter estimates are more precise, due to the need to fit fewer parameters. As survival functions vary over time they should not vary significantly for any unobserved covariates (heterogeneity). When a survival function demonstrates heterogeneity, leading to a poor model fit, then a multiplicative random effect known as the frailty is added to the survival function. By introducing frailty, one may show how the unobserved covariates affect survival. We compare best fitting survival models with and without a parametric frailty term.

### Selection of parametric hazard function

The rate of academic promotion with advancing time in rank should increase, peak, and then more gradually decline as would occur with log-normal, log-logistic, and gamma hazard functions [51–55]. Both the gamma and inverse-Gaussian distributions are natural choices for frailty functions, as they are highly flexible, non-negative, and computationally efficient [55]. The best fit of hazard functions, based on the smallest values of the AIC [51–55], was seen with the log-logistic functions with two frailty distributions (see Table 2). The gamma frailty distribution was selected, because unlike the inverse gaussian frailty distribution it converged when all 14 variables were included in the regression model. An advantage of the log-logistic distribution is that its coefficients, β_i_, can be used to calculate time ratios as e^βi^, where the time ratio is the ratio of the time after a one-unit increment of the variable with a ratio less than one indicating faster promotion [53, 54]. Stata’s stmp2 command was used to draw parametric survival analysis graphs [56, 57].

**Table 2:** Selection of hazard function for parametric survival analysis.

ASSOCIATE PROFESSORS			**Model Size**			
			FULL		SMALL	
Method	Distribution	Frailty	AIC	Vars	AIC	Vars
Cox	PropHaz		9013	12	9009	5
Parametric	Exponential		1711	7	1707	5
	Weibull		-638	13	-641	8
	Gompertz		-215	13	-217	9
	Lognormal		-999	10	-997	5
	GenGamma		†		†	
	Loglogistic	None	-1133	13	-1139	7
yearPhD	Loglogistic	Gamma	-1147	13	-1151	6
	Loglogistic	InvGaussian	-1146	13	-1151	7
FULL PROFESSORS			**Model Size**			
			FULL		SMALL	
Method	Distribution	Frailty	AIC	Vars	AIC	Vars
Cox	PropHaz		5018	13	5012	7
Parametric	Exponential		1056	7	1064	1
	Weibull		-433	12	-433	8
	Gompertz		-229	13	-232	8
	Lognormal		-552	12	-559	7
	GenGamma		-552	12	-558	7
	Loglogistic	None	-656	12	-662	8
	Loglogistic	Gamma	-675	13	-683	8
	Loglogistic	InvGaussian	†		†	

AIC is Akaike information criteria values calculated by Stata’s aic_selection_model with pe(0.8) and pe(0.05), ProHaz is proportional hazards, GenGamma is generalized gamma, and InvGaussian is the inverse gaussian distribution. Full model selected typically starts with 12 variables plus an interaction term, small model contained at least 4 variables plus an interaction term with cumulative time and publications (see next section for explanation).

^†^GenGamma and InvGaussian fails to converge with large and small variable models.

### Choice of incremental and cumulative years and publications

The choice of whether to use time and publication variables calculated on a cumulative basis, or since PhD granting, or incremental basis, since PhD granting or most recent change in rank was done empirically by comparing AIC values of survival analysis models with the results shown Table 3. As Table 2 shows the smaller AIC values with parametric survival analysis with gamma frailty using cumulative years and publications gave a better fit than incremental years and publications and will be used in this paper.

**Table 3:** Comparison of cumulative and incremental publication and time values.

**Associate Professors**			CUMULATIVE	INCREMENTAL
Method	Distribution	Frailty	AIC	AIC
Parametric	Loglogistic	None	-1139	-717
	Loglogistic	Gamma	-1151	-738
	Loglogistic	InvGaussian	-1151	-728
**Full Professors**				
Method	Distribution	Frailty	AIC	AIC
Parametric	Loglogistic	None	-662	77.3
	Loglogistic	Gamma	-683	64.6
	Loglogistic	InvGaussian	†	†

AIC is Akaike information criteria. Log-logistic parametric survival models were selected using Akaike information criteria values calculated by Stata’s aic_selection_model with pe(0.8) and pe(0.05).

^†^ Loglogistic distribution with inverse gamma frailty fails to converge.

### Adjusting the rate and number of publications for research group size

The number and rate of publications for each professor were divided by fractional powers, or k, of the number of authors per paper, or N, with k varying from 0.1 to 0.9. At each fractional power survival analysis using four variables; number and rate of publication, years until hired, and number of moves as an assistant professor; and the interaction of years until hired and rate of publication to estimate AIC values and predict the mean incremental years until promotion using the Stata margins, predict(mean) command.

## Results

### Descriptive statistics

Table 4 summarized the study’s descriptive statistics. Promotion to associate professor after hiring occurs about 8.7 years after PhD granting or about 6.8 years after hiring which is close to 6.9 years expected by institutional tenure policy (see below). The incremental time to promotion to become a full professor, a suspiciously brief 6.7 years, is probably biased due to ending our follow up of professors in 2020 well before many professors would have been promoted. This suggests that professor’s productivity measures are probably biased by the more rapid promotion of highly productive professors whose number and rate of publications as well as citations and size of research group are likely to be greater than those awaiting promotion. The gains for professors with PhDs from top rated departments or by being hired or promoted by top rated departments are modest, 0.2 to 0.4 years. Moves by an assistant professor to another institution delayed their promotion by 0.2 to 0.3 years. About 80% of professors who move to another institution move to a PhD granting department. Most geography professors have degrees from a geography department, about 70%, but the remaining 30% have degrees in environmental sciences, geology, and other disciplines [58]. Women make up no more than a third of professors, suggesting close attention should be directed to the gender coefficient in survival analysis models.

**Table 4:** Descriptive statistics.

		Associate Professor		Full Professor	
		Mean	SD	Mean	SD
Cumulative	Years Until	8.68	2.96	15.4	4.07
Publications	Number	16.1	11.4	34.2	21.4
	Rate	1.95	1.37	2.34	1.54
Citations		446	641	905	1370
Authors		2.85	1.69	3.40	1.90
Top	PhD	0.378	0.485	0.390	0.488
	Assistant	0.202	0.402	0.227	0.419
	Associate	0.177	0.382	0.203	0.402
Moves	Number	0.309	0.536	0.324	0.543
	PhD Granting	0.230	0.421	0.251	0.434
Years Until	Hired	1.94	2.65	2.02	2.78
Year PhD	Granted	1999	5.81	1996	5.51
Gender	Female	0.334	0.475	0.290	0.454
Geography		0.723	0.448	0.706	0.456
N		847		538	

Cumulative years were measured from PhD granting with cumulative publications and citations extending back to five years before PhD granted. Many Assistant Professors were hired the same year as PhD was granted preventing rate of publication from being calculated. Mean of Region is not included, N is number of professors, but excludes professors hired as associate or full professors. Definitions of variables are listed in Table 1.

### Rate and number of publications have opposite effects on promotion

Hypothesis 1 that number and rate of publication have opposite effects on the years until promotion was tested with both small and large survival analysis models as shown in Table 5. For both assistant and associate professor promotion: (1) a greater rate of publication hastens promotion; and (2) promotion is slowed by a greater number of publications, more years between PhD granting and hiring as an assistant professor, more moves as an assistant professor, and the interaction between the rate of publication and years until hired. The interaction between the rate of publication and years until hired shows that at slow rates of publication, such as one publication per year, increasing years until hired as an assistant professor (Year Until Hired) slows promotion; this will be discussed below. As expected, there is strong positive correlation between number and rate of publication which increases the variance inflation factors (not shown), but they remain within the generally acceptable range of less than five [38, 40, 42, 43, see S1 Table in S1 File and related text]. The interaction between the number and rate of publication was not statistically significant and small in magnitude, changing the time ratio less than 0.001. The other significant variables were number of moves as an assistant professor and number of years until hired as an assistant professor; both slowed promotion, and full professor citations hastened promotion but only to a negligible degree. The other variables were not significant including number of authors, affiliation with a top ranked department, moves as an assistant professor to a PhD granting institution, a PhD in geography, year of PhD granting, gender, and region. With parametric survival analysis with gamma frailty, the fit of the small models was not significantly different from that of the larger models as measured by AIC. Last, as measured by Akaike information criteria (AIC), the associate professor models fit better than the full professor models perhaps reflecting the larger sample size in the associate professor model (see Table 5). Note that the better fit after adding gamma frailty to loglogistic parametric survival analysis suggests that the model has missing variables [52–54]. In addition to time ratios, survival analysis can estimate both survival and hazard curves.

**Table 5:** Survival analysis time ratios of variables predicting years to promotion.

Predict Years Until Promotion Using Survival Analysis					
		ASSOCIATE PROFESSOR		FULL PROFESSOR	
SIZE OF REGRESSION MODEL		Large	Small	Large	Small
Independent Variables		Time Ratios		Time Ratios	
Publications	Number	1.043***	1.043***	1.024***	1.025***
	Rate	0.730***	0.729***	0.723***	0.720***
Citations		1.000		0.99998*	0.99999+
Authors		1.002		1.003	
Top	PhD	1.010		1.010	
	Assistant Prof	1.014		0.996	
	Associate Prof			(0.993)	
Moves	Number	1.077***	1.055***	1.020+	1.019+
	to PhD Granting	0.974		0.977+	
Years Until	Hired	1.070***	1.071***	1.034***	1.034***
Year PhD	Granted	1.001	1.001	0.997+	0.998+
Gender	Female	1.003		1.007	
Geography		0.981+		0.976+	0.977+
Region		(1.000)		1.001	
Interaction	Rate of Publication				
	*Years Until Hired	0.975***	0.975***	0.988***	0.988***
AIC		-1147	-1151	-675	-683
N		840	840	515	515

Parametric survival analysis used with a log-logistic distribution with gamma frailty. The dependent variable is time to an event, promotion to become an associate or full professor, measured as years between PhD granting and promotion. Time ratios are shown where values less than one mean that the variable hastens promotion and values greater than one slows promotion. Variables shown in parenthesis were excluded by the automated variable selection algorithm and were calculated using the full model. Cumulative publications include five years before PhD granted. Associate is associate professor, and Professor is full professor, Prof is professor, Years Until Hired is number of years between PhD granting and hiring as an assistant professor, and AIC is Akaike information criteria. Definitions of variables are listed in Table 1. Statistical significance:

^+^ p < 0.05,

* p < 0.01,

** p < 0.005,

*** p < 0.0005

**Fig 1:**
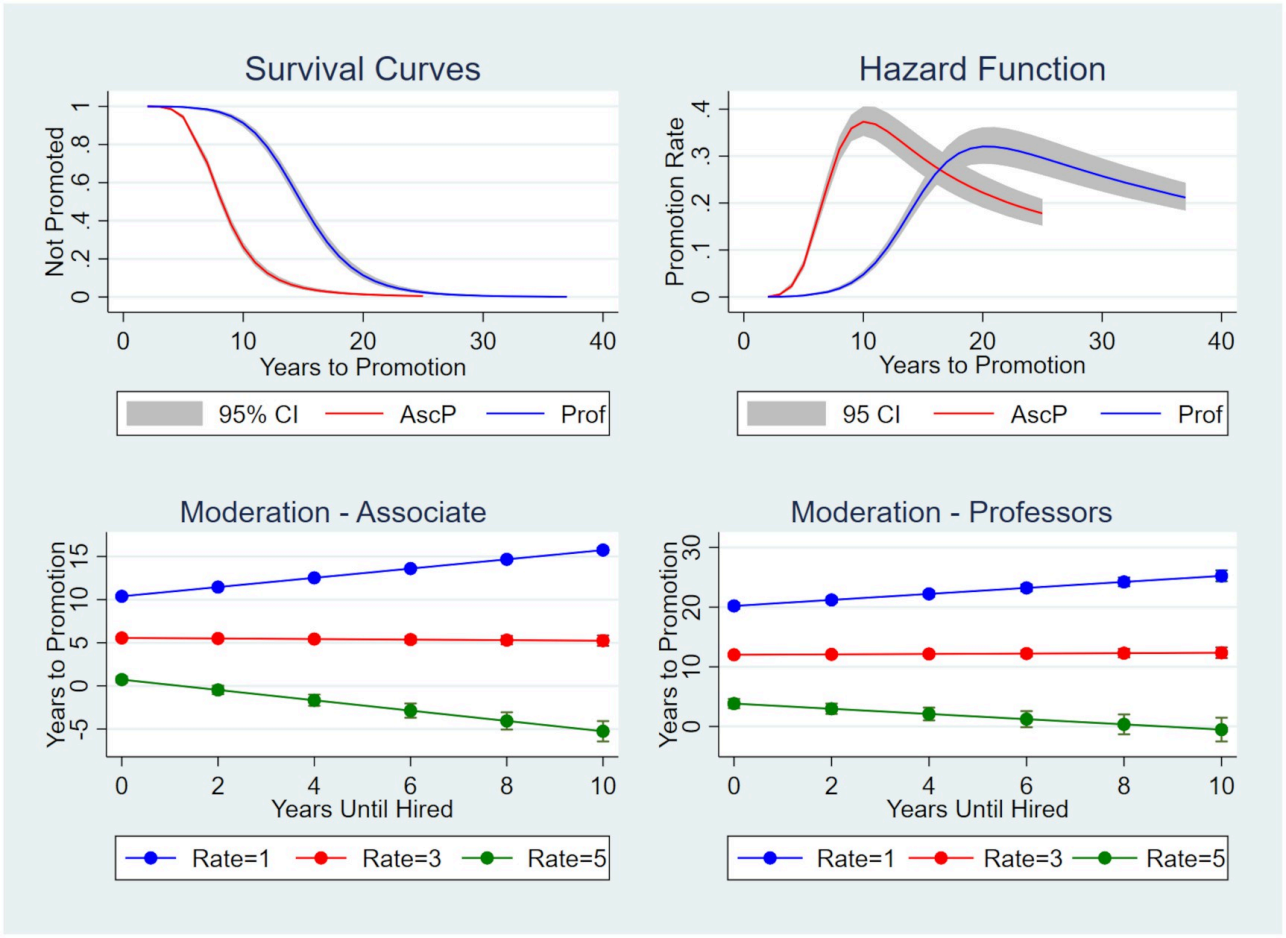
Graphs of hazard and survival curves and interaction. Upper two graphs are: (A) the survival curve of the proportion of professors not promoted vs. years to promotion, and (B) the hazard function showing rate of promotion vs. years to promotion. In both graphs red indicates associate professor (AscP) promotion, and blue full professor (Prof) promotion. The lower two curves show the interaction of years until promoted vs. years until hired as moderated by rate of publication where Rate is number of publications per year for associate professors (C) and full professors (D). Note that a publication rate of five is associated with a negative slope or faster promotion with increasing years until hired, and a publication rate of one is associated with a positive slope or slower promotion. In the upper graphs 95% CI is the 95% confidence level and in the lower graphs the 95% confidence intervals are shown by bars most obvious with Rate = 5 to the right.

Survival and hazard curves are shown in Fig 1. The survival curve, Fig 1A, plots the fraction of professors not promoted vs. years from PhD granting to promotion, and the hazard function, Fig 1B, plots the rate of promotion vs. the years from PhD granting to promotion. Remember that professors on average spend two years between PhD granting and hiring as an assistant professor (see Table 4). The survival curve shows that few become associate professors in less than five years (less than about 10%) but 80% are associate professors after about 11 years. Similarly, less than 10% become full professors within 10 years, but 80% are full professors by about 18 years. The hazard function shows that rate of promotion to become an associate professor peak at about 37% per year after about 10 years with the peak for full professors at about 32% per year after about 21 years; the rate of promotion is based on professors not already promoted. By the time that the rate of promotion peaked, after about 10 years for associate professors and about 21 years for full professors, more than 80% of associate and full professors have been promoted and the promotion rate becomes more variable. The more variable portion of the hazard curves often reflects a small number of professors who had a career in government or industry before gaining an academic position.

### Number and rate of publication increases with increasing research group size

**Fig 2:**
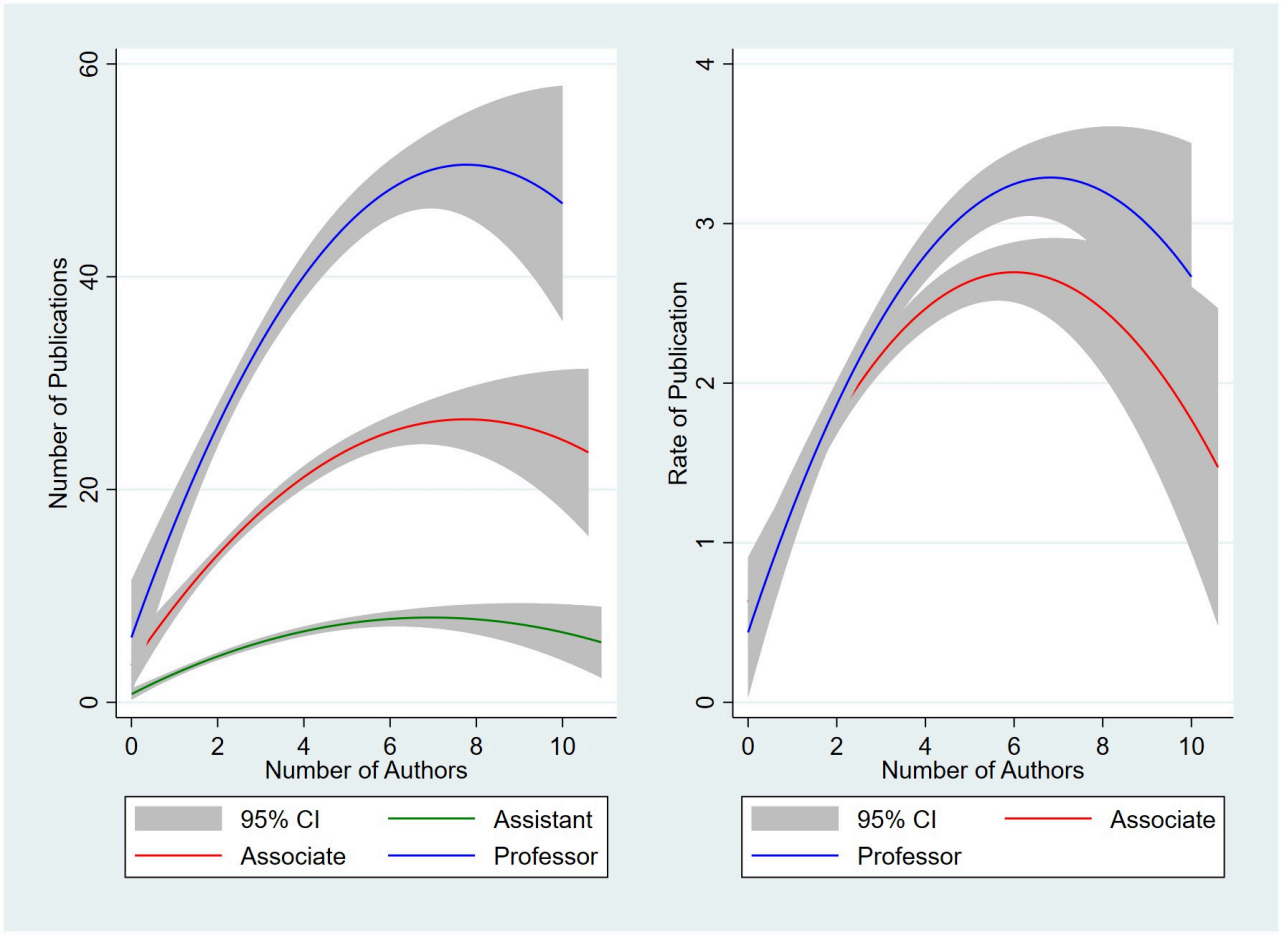
Number and rate of publication vs. number of authors. (A) Number of publications shown on left and (B) rate of publication on right. Assistant professors (Assistant) shown with green lines, associate professors (Associate) with red lines, full professors (Professor) with blue lines, and shaded area is the 95% confidence intervals. Rate of publication not shown for assistant professors as many were hired the same year their PhD was granted forcing division by zero to calculate of the rate of publication.

As research group size increases the rate and number of publications would be expected to increase as proposed by Hypothesis 2 (S2 Table in S1 File and Fig 2). With increasing academic rank, additional authors are associated with significant increases in publications with no overlap in their 95% confidence intervals of their linear coefficients, but not their quadratic terms. For assistant professors, the rate of publication could not be calculated as many professors were hired the same year their PhDs were granted forcing division by zero. For rate of publication for other ranks, both the linear and quadratic coefficients are statistically significant but overlapped for associate and full professors. For the number and rate of publication, the gains with quadratic over linear regression are statistically significant but of minor magnitude with the increase in R^2^ increasing the explained variance by no more than 6% and typically less than 3%. Although the estimated peak values were consistently above six, few professors were members of research groups of six or more, about 4% to 5% for assistant and associate professors and 9% to 10% for full professors. Thus, most professors worked in groups where productivity increased as group size increased, and only a minority worked in large enough groups where group size might reduce productivity.

### Publication rate moderates effect of years to hiring on promotion

Testing Hypothesis 3, the interaction term in the regression model (see Table 5) shows that changing the rate of publication alters, or moderates, the slope of the relationship between years until promoted and years until hired as shown in Fig 1C and 1D. As these graphs show, the relationship between years until promotion to years until hired has a positive slope of about 0.5 for associate and full professors when the rate of publication is one, or Rate = 1, but when Rate = 5 the slope is a negative 0.6 for associate and negative 0.4 for full professors. Relatively few associate professors published at a Rate = 3 or more, about 15%, and about 25% for full professors. Thus, for the same amount of postgraduate experience, professors with a higher rate of publication, such as Rate = 3, are promoted faster than those with a slower rate of publication, such as Rate = 1. This suggests that years of postgraduate experience might influence either the number or rate of publication.

### Greater postgraduate experience increases number, but not rate of publications

**Fig 3:**
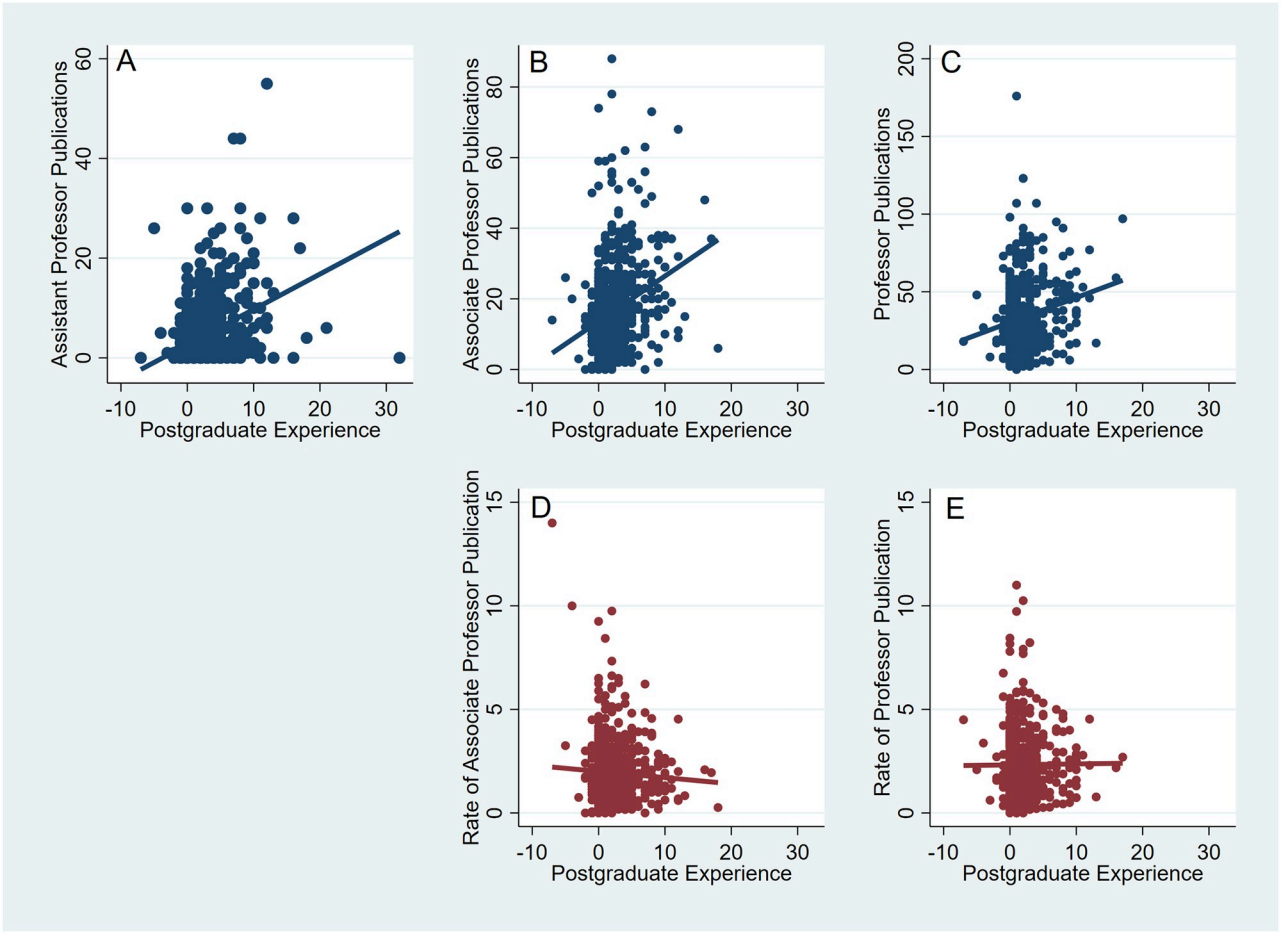
Postgraduate experience increases number, but not rate of publication. Plot of number of publications vs. years of postgraduate experience for assistant professors in A, associate professors in B, and full professors in C. Rate of publication plotted against postgraduate experience for associate professors is shown in D and for full professor in E. Lines are based on linear regression.

Testing of Hypothesis 4 that the number of publications, but not the rate, would increase with increasing postgraduate experience is shown in Fig 3 and S3 Table in S1 File. As Fig 3 shows at all academic ranks the number of publications increases with increasing postgraduate experience, but rate of publication is independent of postgraduate experience. Linear regression coefficients for number of publications vs. postgraduate experience are positive and tend to increase with increasing rank, but coefficients for rate of publication are of small magnitude and not statistically significant. Both rate and number of publications are highly variable and associated with declining adjusted R^2^ values where the explained variance falls from about 15% to less than 5% as academic rank increases. What is clear is that rate of publication is independent of postgraduate experience, while number of publications increases with greater postgraduate experience.

### Postgraduate experience and research group size influence hiring and promotion

Hypothesis 5 was evaluated by dividing professors: (1) with minimal postgraduate experience vs. those with more; and (2) working in small vs. larger research groups as shown in Table 6. About 58% of assistant professors were hired within a year after their PhDs were granted and 54% had an average of two or more authors per paper. When hired the number of publications was about the same in those with a year or more of postgraduate experience, 6.0, as those with two or more members in their research group 5.6 (t-test, p = 0.39, not significant). Those hired with more postgraduate experience, > 1 year, had a smaller research group size than those working in larger research groups of ≥ 2, 2.6 vs. 3.7 (t-test, p< 0.00005). On average professors hired more than a year after their PhD was granted invested more time in postgraduate training, about four years as measured by years until hired. However, if four years invested in postgraduate training are subtracted from their promotion times to associate and full professor they were promoted about a year or two more quickly. When professors working in larger research groups of two or more were compared with those working in smaller research groups the following differences were noted: (1) those in larger groups spent 0.67 years more time in postgraduate training but took only about 0.42 years longer to be promoted to become associate professors, and took the same number of years to become full professors; and (2) gained tenure on average about 6.6 years after being hired which was close to the 6.7 years required by current tenure guidelines (t-test, not significant, see Tenure section). At all academic ranks professors who had either more experience, or more authors when hired: (1) moved less often as assistant professors, (2) had larger research groups, and (3) had more publications when promoted. However, a greater rate of publication was only seen in professors who were members of larger research groups and not in professors hired with more experience. Given the relatively small advantage of professors working in larger vs. smaller research groups it is possible that promotion committees adjust number and rate of publication for research groups size to increase fairness in promotion.

**Table 6:** Professors dichotomized by postgraduate experience and authors.

	Years Until Hired			Authors When Hired		
ACADEMIC RANK	≤ 1	> 1	Diff	< 2	≥ 2	Differ
**Assistant** (N and %)	579 (58%)	422 (42%)		455 (46%)	546 (54%)	
Years Until Hired	0.261	4.227	****	1.569	2.236	***
Number of Publications	2.572	5.979	****	2.084	5.612	****
Moves as Assistant Professor	0.356	0.219	****	0.345	0.259	*
Number of Authors	1.973	2.602	****	0.541	3.652	****
**Associate** (N and %)	477 (56%)	369 (44%)		381 (45%)	465 (55%)	
Years to Promotion	7.478	10.21	****	8.436	8.858	+
Number of Publications	13.77	19.12	****	11.99	19.48	****
Rate of Publication	1.962	1.936		1.555	2.275	****
Number of Authors	2.485	3.328	****	2.015	3.652	****
**Professor** (N and %)	285 (55%)	230 (45%)		242 (47%)	273 (53%)	
Years to Promotion	14.09	16.43	****	15.22	15.06	ns
Number of Publications	30.20	37.57	***	26.57	39.62	****
Rate of Publications	2.285	2.389		1.872	2.738	****
Number of Authors	2.879	3.911	****	2.491	4.093	****

Diff is difference, Assistant is assistant professor, Associate is associate professor, and Professor is full professor. Means are shown. Number of professors (N) declines with advancing rank but proportions (%) in each group are similar. Significance of t-test: ns not significant,

^+^ p < 0.05,

* p < 0.01,

** p < 0.005,

*** p < 0.0005, and

**** p < 0.00005.

### Promotion committees adjust productivity for research group size, but effect negligible

**Fig 4:**
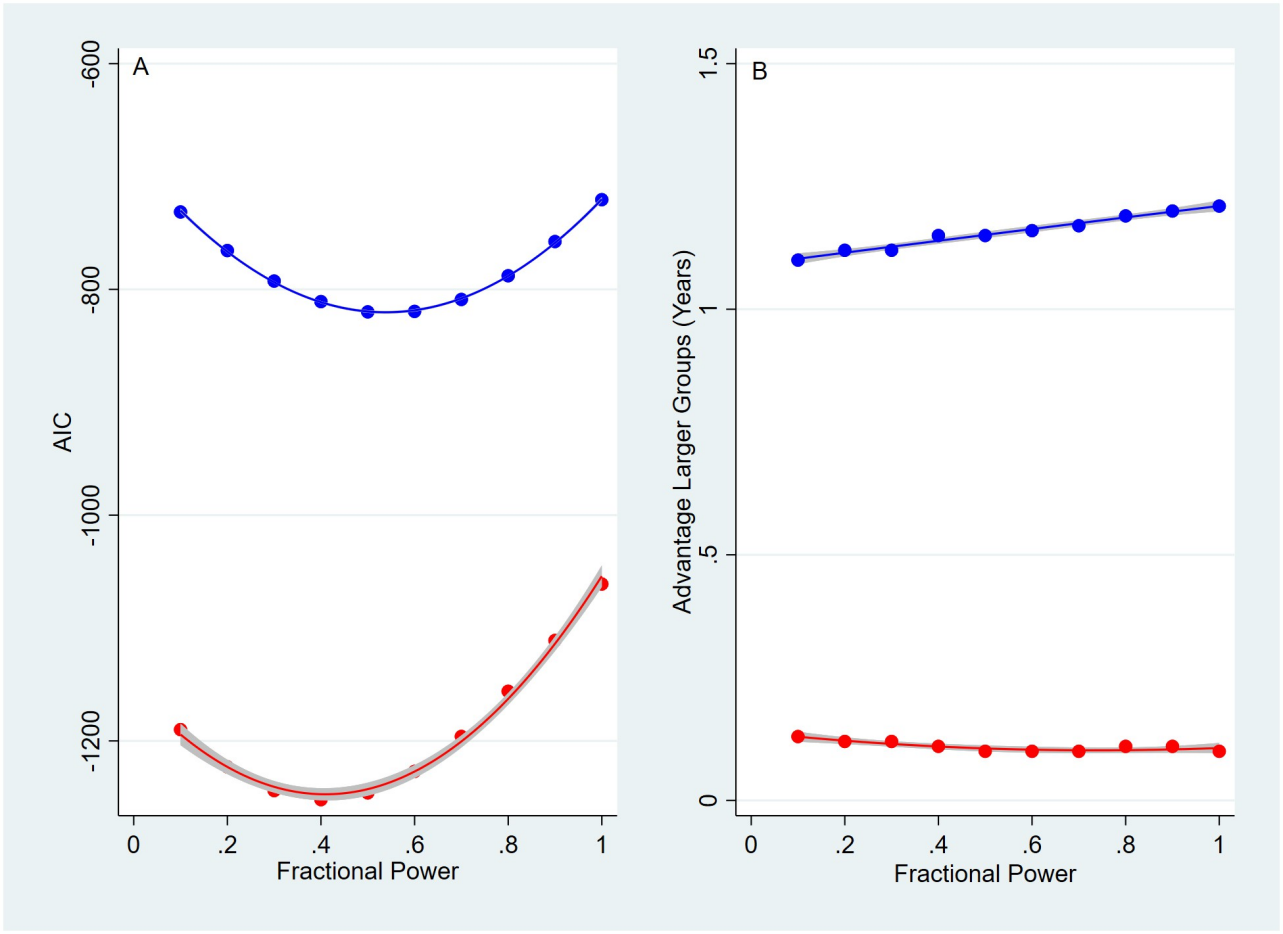
AIC values and advantage large research group vs. fractional power. Parametric survival analysis with a log-logistic distribution and gamma frailty used the small model (the four highly significant variables and interaction term shown in S4 Table in S1 File) with number and rate of publication divided by the fractional powers (k) of the number of authors, or N^k^, with k varying from 0.1 to 1 in steps of 0.1. Remember a fractional power of zero is the same as dividing by one which is the average value. On the left, see A, the AIC values associated with each fractional power are plotted. The minimum AIC value occurs at a fractional power of about 0.41 for associate professors and about 0.54 for full professors. On the right, see B, is the average advantage in years of being a member of a larger vs. small research group as estimated with survival analysis. The average of the number of authors per publication was used to create larger and smaller research groups used 2.85 for associate professors and 3.38 for full professors. Associate professors are shown in red, full professors in blue, the fitted line is a quadratic in A and for associate professors in B with a linear fit for full professors in B. The 95% confidence intervals are shaded.

Promotion committees would be expected to adjust a professor’s number and rate of publication for her research group size. Hypothesis 7 was tested by dividing the number and rate of publication by research group size (N) or a fractional powers of research group size, N^k^, with k varying from 0.1 to 1 in steps of 0.1. Using parametric survival analysis with a log-logistic distribution and gamma frailty, the smallest AIC values occurred when publications are divided by k = ~ 0.41 for associate and ~ 0.54 for full professors (see Fig 4 and S4 Table in S1 File). This shows that promotion committees have chosen a plausible midpoint in the range of fractional powers. To divide by the total number of authors, N, would give no advantage over working alone. Similarly, smaller powers of N such as 0.1 might give an undue advantage to collaboration over individual work. These results are approximately those expected by the so called Price’s square- root law and Bikard, Murray, and Gans report of an empirically derived fractional power of 0.48 for the allocation of credit for citations to individual members of a research group [12, 58, 59]. Our results are similar and suggest that promotion committees do consider research group size when making promotion decisions.

However, as Fig 4B shows, the actual effect of correcting for different research group sizes is slight for associate professors and small for full professor. First, the relationship between years to promotion to research group size is insignificant for associate professors and small but significant for full professors (linear regression coefficients for incremental years to promotion vs. research group size, slope -0.225, p < 0.001, for full professors). Next, as measured by the mean incremental years to promotion, the advantage for professors working in large groups over those working in small groups only averaged 0.13 years for associate professors, but was nearly a year, or 0.98 years, for full professors. Similar results were found when survival analysis was used to estimate the average advantage in incremental years to promotion of professors working in larger research groups over those working in smaller research groups with the advantage varying from 0.10 years to 0.13 years for associate professors vs. 1.10 to 1.21 years for full professors (see S5 Table in S1 File and related text). Note that any advantage of one fractional power over another was slight– 0.03 years for associate professors, but a slightly larger 0.11 years for full professors. Even the quadratic relationship for AIC and fractional powers as clearly shown in Fig 4A is muted in 4B. In Fig 4B the advantage of working in a larger vs. smaller research group is quadratic for associate professors, but linear for full professors (see S6 Table in S1 File and related text). The results show that promotion committees tend to promote professors from assistant to associate professors after about 6.8 to 6.9 years with differences based on research group size being slight, while promotion from associate to full professor varying much more, about 6.4 vs. 7.5 years, with those working in smaller research groups taking about a year longer to be promoted. Why the promotion to full professor is a year faster for professors working in larger research groups is unclear, but tenure policy may explain why associate professor promotion is virtually independent of their research group size.

### Tenure clock stable over thirty years

Newly hired assistant professors serve a probationary period before they are considered for promotion to associate professor when they typically gain tenure. Hypothesis 7 was tested by reviewing the tenure policy of the 74 institutions making up this study in 2021 showed that 1 institution (1.4%) left tenure policy to the department and the rest used the following times: (1) 5.5 years in 1 institution or 1.4%; (2) 6 years in 27 or 36.5%; (3) 7 years in 42 or 56.8%; (4) 8 years in 2 or 2.7%; and (5) 10 years in 1 or 1.4%. Unfortunately, the professors in this study were identified between 1990 and 2010 when the tenure policy might have been different. This was tested by calculating the tenure time as the time from hiring as an assistant professor to the time to promotion to become an associate professor after excluding assistant professors who moved to another institution before they were promoted, as moves were likely to alter the tenure clock. The mean time to tenure in the 74 institutions was 6.12 years ± 0.0943 (mean ± standard error) compared to the 2021 tenure guidelines of 6.68 years ± 0.0789; this difference was significant, p < 0.00005. Since tenure policy provided an upper limit, the results show that the tenure clock has been relatively stable for about 30 years. Most professors, about 35%, gained tenure within a year of that specified by their university’s tenure policy with about 16% before and 14% after. Of the remaining 35%, about 17% left their academic job, and 18% moved to another institution. The results are consistent with the idea that tenure policy largely dictates the timing of associate professor promotion.

## Discussion

Like other studies, we found that: (1) academic hiring and promotion decisions are dominated by the number of publications a professor has published [23, 25, 27, 30–32]; (2) both the number and rate of publication increase as research group size increases [12, 18, 20, 24, 28]; and (3) postgraduate experience influences a professor’s career [25, 31]. What was unexpected, and possibly novel, is that rate and number of publications had opposite effects on cumulative time to promotion measured from PhD granting with rate of publication hastening promotion and number of publications slowing promotion. Statistically this effect reflects the fact that number and rate of publication are reciprocal suppressor variables [see S1 Table in S1 File and related text, 40–46]. A more intuitive explanation is that professors with more postgraduate experience tended to work in smaller research groups, published more slowly, and needed more postgraduate experience to publish the required number of publications to be hired as an assistant professor. This would tend to associate more publications with a longer time to hiring and promotion if time to promotion is measured from PhD granting. Conversely, we observed that professors who worked in larger research groups produced the required number of publications to be hired and promoted more quickly than those working in small research groups. This tended to associate rapid rates of publication with faster promotion. In sum, professors who work in smaller (larger) research groups, publish more slowly (rapidly), have more (less) postgraduate experience, and take more (less) time to be hired and promoted if time to hiring and promotion is measured from PhD granting. Unsurprisingly, survival analysis models fit much better when cumulative time from PhD granting than then when incremental years from hiring or most recent promotion were used. Institutional tenure policy dictates the timing of tenure and typically promotion to become an associate professor and explains why promotion decisions are made a fixed number of years after hiring.

Tenure and promotion committees must follow institutional policies dictating that tenure decisions typically be made within six to seven years after hiring as an assistant professor and similar, less strict, guidelines may also apply to promotion to become a full professor [6, 7, 21–27]. Tenure and promotion committees measure time to promotion from hiring or most recent promotion, not from PhD granting, and as a result professors with more postgraduate experience are promoted after about the same number of incremental years as those working in larger research groups. The rate of promotion, as shown in hazard curves, is rapid during the six-to-seven-year time window required by institutional tenure policy; survival curves show similar results as do other studies [25, 51, 52, 59]. The timing of tenure decisions is dictated by institutional policy, but do promotion committees consider research group size in making their decisions?

We investigated whether promotion committees adjust measures of productivity, the number and rate of publication, by dividing them by a fractional power of the number of authors or research group size. Bikard, Murray, and Gans studied the allocation of credit for citations by dividing the number of citations by the size of a professor’s research group, N, or a fractional power (k) of N, or N^k^ which they varied from 0.1 to 1 [12]. Their idea was that if citations were divided by the total number of authors, or N, there would be no incentive to collaborate and if the citations were divided by N^0.1^ then collaboration would be favored too strongly [12]. Their empirical result was that citations were divided by N^0.48^ [12]. Similarly, credit for publications might follow Price’s square-root law where contributions to the literature follow the square root of the authors in a field [48, 49]. We used Akaike information criteria (AIC) to measure model fit of our survival analysis model as we divided the number and rate of publications by fractional powers of the number of authors which varied from 0.1 to 1 and found that the best fit when the number and rate of publications were divided by the number of authors raised to the ~0.41 power for associate professor, and by the ~0.54 power for full professor promotion. However, we were surprised that for associate professor promotion there was no advantage for working in a larger vs. smaller research group, with differences measured in 0.1s of a year, although full professors working in larger groups were promoted a year more quickly than those working in a small research group. It appears that institutional tenure policy may equalize associate professor promotion so that research group size has a negligible effect. Less clear is why full professors working in larger research groups are promoted a year more quickly. The better fit of our survival analysis models with the use of gamma frailty suggests that our regression models lack significant variables, such as teaching, grant funding, or administrative experience [52–54]. Rahmandad and Vakili [9] present evidence that research funding encourages professors to shift from working in small research groups to working in larger and more productive research groups. We suspect that a combination of tenure policy and grant funding might explain the differences in years to promotion for small vs. larger research groups.

Before students and their advisors use the results to help plan their career pathway, some limitations of this study should be considered. These limitations include: (1) the lack of information about how hiring and promotion committees make their decisions [47]; (2) the limited ability of research group size to explain the number and rate of publication [11, 13, 24]; (3) the lack of evidence that promotion committees actually adjust the number and rate of publication for research group size vs. simply following tenure and promotion policies [12, 24, 28]; (4) the lack of information about a professor’s service, teaching, administrative responsibilities and research grant funding [9, 15, 21, 25, 27–29]; (5) the limited analysis of differences between institutions [11, 13]; and (6) the narrow focus on PhD granting departments of geography. Many of these topics invite further study. Given the overlap of geography with other social sciences it would be expected that our results would extend to other social sciences [58]. Despite these limitations, this study does provide more information concerning at least two paths that lead to a successful academic career and some of the trade-offs with each.

## Supporting information

S1 File (DOCX)

S1 Data (XLSX)

## References

[1] Peet RK, Glenn-Lewin DC, Stearns F, Kangas PC. The Academic Job Market for Ecologists: Observations from Four Search Committees. Bull. Ecol. Soc. Am. 1985; 66: 2–5.

[2] Sauermann H, Roach M. Why pursue the postdoc path? Complex, diverse rationales require nuanced policies. Science. 2016; 352: 663–4. 27151854 10.1126/science.aaf2061

[3] Seager J. “And a Charming Wife”: Gender, Marriage, and Manhood in the Job Search Process. Prof. Geogr. 2000; 52: 709–21.

[4] Coggburn JD, Neely SR. Publish or perish? Examining Academic Tenure Standards in Public Affairs and Administration Programs. J. Pub. Aff. Educ. 2015; 21: 199–214.

[5] Ferrer RL, Katerndahl DA. Predictors of Short-term and Long-term Scholarly Activity by Academic Faculty: A Departmental Case Study. Fam. Med. 2002; 34: 455–61. 12164624

[6] Gibbs P, Locke B. Tenure and Promotion is Accredited Graduate Social Work Programs. J. Soc. Work Educ. 1989; 25: 126–33.

[7] Marshall BW, Rothgeb JM Jr. So You Want Tenure? Factors Affecting Tenure Decisions in Political Science Departments. PS: Polit. Sci. Polit. 2011; 44: 571–7.

[8] Pennycook G, Thompson VA. An Analysis of the Canadian Cognitive Psychology Job Market (2006–2016). Can. J. Exp. Psychol. 2018; 72: 71–80. doi: 10.1037/cep0000149 29902028

[9] Rahmandad H, Vakili K. Explaining Heterogeneity in the Organization of Scientific Work. Organ. Sci. 2019; 30: 1125–45. doi: 10.1287/orsc.2019.1303

[10] Ross RG, Greco-Sanders L, Laudenslager M, Reite M. An Institutional Postdoctoral Research Training Program: Predictors of Publication Rate and Federal Funding Success of Its Graduates. Acad. Psychiatry. 2009; 33: 234–40. doi: 10.1176/appi.ap.33.3.234 19574523 PMC2705878

[11] Long JS, McGinnis R. Organizational Context and Scientific Productivity. Am. Sociol. Rev. 1981; 46: 422–42.

[12] Bikard M, Murray F, Gans JS. Exploring Trade-offs in the Organization of Scientific Work: Collaboration and Scientific Reward. Manage. Sci. 2015; 61: 1473–95. doi: 10.1287/mnsc.2014.2052

[13] Clauset A, Arbesman S, Larremore DB. Systematic inequality and hierarchy in faculty hiring networks. Sci. Adv. 2015; 1: e1400005. doi: 10.1126/sciadv.1400005 26601125 PMC4644075

[14] Holland TL, Keewan K, Nobles CJ, Lu Y-L, Seeni I, Mumford SL, et al. Length of Fellowship Training in Population Health Research and Long-term Bibliometric Outcomes. Epidemiol. 2019; 30: S85–S93. doi: 10.1097/EDE.0000000000001093 31569157 PMC8312074

[15] Taylor JS, Friedman RH, Speckman JL, Ash AS, Moskowitz MA, Carr PL. Fellowship Training and Career Outcomes for Primary Care Physician-Faculty. Acad. Med. 2001; 76: 366–72. doi: 10.1097/00001888-200104000-00015 11299152

[16] Yang L, Webber KL. A decade beyond the doctorate: the influence of a US postdoctoral appointment on faculty career, productivity, and salary. High. Educ. 2015; 70: 667–87.

[17] Wuchty S, Jones BF, Uzzi B. The Increasing Dominance of Teams in Production of Knowledge. Science. 2007; 316: 1036–39. doi: 10.1126/science.1136099 17431139

[18] Bozeman B, Youtie J. The Strength in Numbers: The New Science of Team Science. Princeton: Princeton University Press; 2017.

[19] Katz JS, Martin BR. What is research collaboration? Res. Policy. 1997; 26: 1–18.

[20] Leahey E. From Sole Investigator to Team Scientist: Trends in the Practice and Study of Research Collaboration. Annu. Rev. Sociol. 2016; 42: 81–100. doi: 10.1146/annurev-soc-081715-074219

[21] Abeles H, Doyle A. The Promotion and Tenure Process in CMS Members’ Music Units. Coll. Music Sym. 2018; 58: 1–22.

[22] Faria JR, McAdam P. Academic productivity before and after tenure: the case of the ’specialist’. Oxf. Econ. Pap. 2015; 67: 291–309.

[23] Hesli VL, Lee JM, Mitchell SM. Predicting Rank Attainment in Political Science: What Else Besides Publications Affects Promotion? PS Polit. Sci. Polit. 2012; 45: 475–92. doi: 10.1017/S1049096512000364

[24] Lee S, Bozeman B. The Impact of Research Collaboration on Scientific Productivity. Soc. Stud. Sci. 2005; 35: 673–702.

[25] Long JS, Allison PD, McGinnis R. Rank Advancement in Academic Careers: Sex Differences and the Effects of Productivity. Am. Sociol. Rev. 1993; 58: 703–22.

[26] Manchester CF, Leslie LM, Kramer A. Is the Clock Still Ticking? An Evaluation of the Consequences of Stopping the Tenure Clock. Ind. Labor Relat. Rev. 2013; 66: 3–31.

[27] National Research Council. From Scarcity to Visibility: Gender Differences in the Careers of Doctoral Scientists and Engineers. Washington: National Academies Press; 2001.

[28] Hollis A. Co-authorship and the output of academic economists. Labour Econ. 2001; 8: 503–30.

[29] Liner GH, Sewell E. Research requirements for promotion and tenure at PhD granting departments of economics. Appl. Econ. Lett. 2009; 16: 765–8. doi: 10.1080/13504850701221998

[30] Bayer AE, Astin HS. Sex differentials in the academic reward system: What changes have there been since the implementation of federal antibias regulations? Science. 1975; 188: 796–802. 17769878 10.1126/science.188.4190.796

[31] Ginther DK, Hayes KJ. Gender Differences in Salary and Promotion for Faculty in the Humanities 1977–95. J. Hum. Resour. 2003; 38: 34–73.

[32] Ginther DK, Kahn S. Does science promote women? Evidence from academia 1973–2001. 2006. https://www.nber.org/chapters/c11621.pdf.

[33] Ceci SJ, Ginther DK, Kahn S, Williams WW. Women in Academic Science: A Changing Landscape. Psychol. Sci. 2014; 15: 75–141. doi: 10.1177/1529100614541236 26172066

[34] Hargens LL. Academic Labor Markets and Assistant Professors’ Employment Outcomes. Res. High. Educ. 2012; 53: 311–24. doi: 10.1007/s11162-011-9228-1

[35] Seggie SH, Griffith DA. What Does It Take to Get Promoted in Marketing Academia? Understanding Exceptional Publication Productivity in the Leading Marketing Journals. J. Mark. 2009; 73: 122–32.

[36] McElrath K. Gender, Career Disruption, and Academic Rewards. J. Higher Educ. 1992; 63: 269–81.

[37] Benavides S, Garcia AS, Caballero J, Wolowich WR. The Impact of Student-Faculty Ratio on Pharmacy Faculty Scholarship. Am. J. Pharm. Ed. 2010; 78 Article 138. doi: 10.5688/aj7408138 21179249 PMC2987278

[38] Lam A, Heslin MJ, Tzeng C-WD, Chen H. The Effect of Tenure and Promotion on Surgeon Productivity. J Surg. Res. 2018; 227:67–71. 29804864 10.1016/j.jss.2018.02.020

[39] Allison PD and Long JS. Departmental Effects on Scientific Productivity. Am. Sociol. Rev. 1990; 55: 469–478.

[40] Beckstead JW. Isolating and Examining Sources of Suppression and Multicollinearity in Multiple Linear Regression. Multivar. Behav. Res. 2012; 47: 224–46. doi: 10.1080/00273171.2012.658331 26734849

[41] Cohen J, Cohen P, West SG, Aiken LS. Applied Multiple Regression/Correlation Analysis for the Behavioral Sciences. 3rd ed. New York: Routledge; 2015.

[42] Lenz G, Sahn A. Achieving Statistical Significance with Control Variables and without Transparency. Polit. Anal. 2020; 29: 356–69. 10.1017/pan.2020.31

[43] Meyers LS, Gamst G, Guarino AJ. Applied Multivariate Research: Design and Interpretation. Thousand Oaks: Sage Publications; 2006.

[44] Pandey S, Elliott W. Suppressor Variables in Social Work Research: Ways to Identify in Multiple Regression Models. J. Soc. Social Work Res. 2010; 1: 28–40.

[45] Tabachnick BG, Fidel LS. Using Multivariate Statistics. 7th ed. Upper Saddle River: Pearson; 2020.

[46] Horst P, Wallin PC, Guttman LB, Wallin FB, Clausen JA, Reed RC, et al. The prediction of personal adjustment: A survey of logical problems and research techniques, with illustrative application to problems of vocational selection, school success, marriage, and crime. New York: Social Science Research Council; 1941, p434.

[47] Franke AH. Making Defensible Tenure Decisions. Academe. 2001; 87: 32–6.

[48] Acock AC. A Gentle Introduction to Stata. 6ed. College Station: Stata Press; 2018.

[49] Kaminski D, Geisler C. Survival Analysis of Faculty Retention in Science and Engineering by Gender. Science. 2012; 335: 864–6. doi: 10.1126/science.1214844 22344445

[50] Box-Steffensmeier JM, Cunha RC, Varbanov RA, Hoh YS, Knisley ML, Holmes MA. Survival analysis of faculty retention and promotion in the social sciences by gender. PLoS ONE. 2015; 10(11): e0143093. doi: 10.1371/journal.pone.0143093 26580565 PMC4651362

[51] Hosmer DW, Lemeshow S, May S. Applied Survival Analysis: Regression Modeling of Time-to-Event Data. 2nd ed. Hoboken: Wiley; 2008.

[52] Klein JP, Moeschberger ML. Survival Analysis: Techniques for Censored and Truncated Data. 2nd ed. New York: Springer; 2003.

[53] Wienke A. Frailty Models in Survival Analysis. Chapman and Hall. CRC. New York: Routledge; 2010.

[54] Royston P, Lambert PC. Flexible Parametric Survival Analysis Using Stata: Beyond the Cox Model. College Station: Stata Press; 2011.

[55] Royston P, Parmar MKB. Flexible parametric proportional-hazards and proportional-odds models for censored survival data, with application to prognostic modelling and estimation of treatment effects. Statist. Med. 2002; 21: 2175–97. doi: 10.1002/sim.1203 12210632

[56] Foote KE, Solem MN. Toward better mentoring for early career faculty: results of a study of US geographers. Int. J. Acad. Res. Dev. 2009; 14: 47–58. doi: 10.1080/13601440802659403

[57] Ginther DK, Kahn S. Women in Economics: Moving Up or Falling Off the Academic Career Ladder? J Econ. Perspect. 2004; 18: 193–214.

[58] Ingram AG, Graves J, Peckham V. The Ringelmann Effect: Studies of Group Size and Group Performance. J Exp Soc Psychol. 1974; 10: 371–84.

[59] Nicholls PT. Price’s square root law: Empirical validity and relation to Lakota’s law. Inf Process Manag. 1988; 24: 469–77.

