# Supplementary material for: Rate of publication hastens, but number of publications slows academic promotion
Source: PLoS One. 2022 Oct 26;17(10):e0276616. doi: 10.1371/journal.pone.0276616 (PMC9604874; doi:10.1371/journal.pone.0276616)
Supplement: S1 File — (DOCX) [file pone.0276616.s001.docx]

Supporting information

Rate of Publication Hastens, But Number of Publications Slows Academic Promotion

Jackson B. Pickett MD*, Paul Savala PhD

*Corresponding author. Email: [jpickett@stedwards.edu](mailto:jpickett@stedwards.edu)

This file includes:

Supplementary Text with S1 to S6 Tables

S1 Data File

**Supplementary Text**

**Suppressor Variables**

When originally introduced by Horst, suppressor variables were defined as variables with zero correlation with the outcome variable, yet whose inclusion in a regression equation significantly improved the predictions of the model [1]. Since the introduction of suppressor variables their definition has been expanded. Notably, Conger defines a suppressor variable as “a variable which increases the predictive validity of another variable by its inclusion in a regression equation.” He notes that “a suppressor is not defined by its own regression weight but rather by its effects on other variables in a regression system” [2].

Consider the case of predictor variables X_1_ and X_2_ and an outcome variable Y. Often it is the case that X_1_ and X_2_ are at least somewhat correlated. In this case one would expect the predictive power (measured by R^2^) of the full linear model $Y=\alpha+\beta_{1}X_{1}+\beta_{2}X_{2}+\epsilon$ to be less than the sum of the predictive power of the models $Y=\alpha+\beta_{1}X_{1}$ and $Y=\alpha+\beta_{2}X_{2}$. Suppose, however, that when added to a linear regression model $Y=\alpha+\beta_{1}X_{1}$ to create $Y=\alpha+\beta_{1}X_{1}+\beta_{2}X_{2}$, the predictive power *increases* significantly and the coefficient $\beta_{1}$ also increases significantly. In this case, X_2_ is a called a suppressor variable for the variable X_1_. Similarly, if X_1_ has the same effect when added to $Y=\alpha+\beta_{2}X_{2}+\epsilon$ then X_1_ and X_2_ are called reciprocal suppressor variables [1-8]. This phenomenon is explained by the suppressor variable directly explaining a portion of the error $\epsilon$ in the original linear model. That is, the inclusion of the suppressor variable can directly explain the error from the original model. In this way it “suppresses some outcome-irrelevant variation or error in one or more other predictors” [3].

Notably, suppressor variables may be identified when: (1) the standardized coefficient of X_1_ when X_2_ is present is larger than either the correlation of X_1_ with Y or the standardized coefficient of X_1_ alone predicting Y; (2) there is a significant increase in the regression model fit as estimated by R^2^ when the fit with X_1_ or X_2_ alone are compared to when both are included [4-8].

**Rate and Number of Publications Are Reciprocal Suppressor Variables**

When predicting the years until promotion to become an associate or full professor using linear regression, the number and rate of publication had opposite effects with number of publications slowing promotion and rate of publications hastening promotion (see S1 Table). S1 Table shows standardized linear-regression coefficients relating either number or rate of publications or both combined as independent variables used to predict years to promotion for associate and full professors. When the number and rate of publications are combined the magnitude of their coefficients for both associate and full professor promotion at least doubles, R^2^ increases several-fold, and the root-mean-square error (not shown) decreases by about 30% or more. The magnitude of both standardized and unstandardized (not shown) coefficients increased significantly (p < 0.00005) when a second variable was added to the regression equation, for example for number of associate professor publications from 0.235 to 1.101; this was also true when the first order correlation was compared with the matching two variable coefficient, or 0.241 with 1.104. Also, the first order correlations, FOC in S1 Table, are much smaller than the correlation between number and rate of publication (0.78 for associate professors and 0.85 for full professors); these differences are significant for publications with p < 0.00005. As expected, the strong correlation between number and rate of publication increased the variance inflation factor (VIF). Last, the increase in model fit when both number and rate of publication were compared with either variable alone using nested linear regression was highly significant. In sum, the evidence that number and rate of publication or articles are reciprocal suppressor variables includes: (1) comparison of regression coefficients for one vs. two of variable models; (2) the comparison of first order correlations with standardized two-variable coefficients; and (3) the large change in R^2^ when nested regression was used to compare one vs. two variable models.

**S1 Table. Influence of productivity on years to promotion in nested linear regression model.**

| **Associates** | | COEFFICIENTS | | | FOC | VIF | R^2^ | | ΔR^2^ |
| --- | --- | --- | --- | --- | --- | --- | --- | --- | --- |
| Pub | Num | 0.235  *** |  | 1.101  *** | 0.239 | 2.60 | 0.0579 | 0.524 | 0.467 |
|  | Rate |  | -0.241  *** | -1.104  *** | -0.241 |  | 0.056 |  | 0.467 |
| **Professors** | |  |  |  |  |  |  |  |  |
| Pub | Num | 0.108+ |  | 1.282  *** | 0.116 | 3.55 | 0.012 | 0.553 | 0.542 |
|  | Rate |  | -0.300  *** | -1.386  *** | -0.300 |  | 0.090 |  | 0.463 |

Standardized coefficients are from nested linear regression, FOC is the first order correlation between number or rate of publication and years to promotion, VIF is the variance inflation factor, R^2^ is the correlation coefficient squared, ΔR^2^ is change in R^2^, Pub is publications, Num is number, and Rate is rate of publication. First column under Coefficients shows results with number of publications alone, second column shows rate of publication alone, and third column shows coefficients when both number and rate are combined. Years are counted from PhD granting, and publications were counted from five years before PhD granting. All ΔR^2^ with nested regression have p < 0.00005. Significance: + p < 0.05, * p < 0.01, ** p < 0.005, and *** p < 0.0005.

**S2 Table. Quadratic regression for publications vs. author number.**

|  |  | **Coefficients** | | **Quad** | **Peak** | **R^2^** | | |
| --- | --- | --- | --- | --- | --- | --- | --- | --- |
| **Variable** | **Rank** | Linear | Quad | **Better** | **Authors** | Linear | Quad. | Differ |
| Number | Assistant | 1.781  *** | -0.101  *** | ******* | 8.82 | 0.128 | 0.185 | 0.0566 |
|  | Associate | 6.092  *** | -0.410  *** | ******* | 7.43 | 0.181 | 0.204 | 0.0224 |
|  | Professor | 10.941  *** | -0.659  *** | ******* | 8.30 | 0.226 | 0.249 | 0.0224 |
| Rate | Associate | 0.648  *** | -0.0512  *** | ******* | 6.33 | 0.0909 | 0.115 | 0.0237 |
|  | Professor | 0.791  *** | -0.0554  *** | ******* | 7.14 | 0.152 | 0.181 | 0.0289 |

Nested linear regression was used. Quad is quadratic coefficient, Quad Better is the significance of the comparison between the linear and quadratic fit, Peak Authors were calculated as the [(linear coefficient)/(-2*(quadratic coefficient))]. R^2^ differ is the difference between linear and quadratic regression R^2^ values, Number is number of publications and Rate is rate of publication. Note, rate of publication is not shown for assistant professors as many were hired the same year their PhD was granted forcing division by zero to calculate of the rate of publication, Assistant is assistant professor, Associate is associate professor, Professor is full professor. Significance: + p < 0.05, * p < 0.01, ** p < 0.005, and *** p < 0.0005.

**S3 Table. Relation of number and rate of publication to postgraduate experience**.

| Rank | Publications | Coefficient | aR^2^ | RMSE | Mean | N |
| --- | --- | --- | --- | --- | --- | --- |
| Assistant | Number | 0.709*** | 0.146 | 4.932 | 4.008 | 1001 |
| Associate | Number | 1.284*** | 0.0879 | 10.91 | 15.95 | 900 |
| Professor | Number | 1.595*** | 0.0418 | 20.68 | 33.70 | 564 |
| Associate | Rate | -0.0309 | 0.0024 | 1.366 | 1.927 | 898 |
| Professor | Rate | 0.00453 | -0.0019 | 1.544 | 2.342 | 563 |

Coefficient is linear regression coefficient, aR^2^ is adjusted R^2^, RMSE is root mean square error of linear regression model. Statistical significance: + p < 0.05, * p < 0.01, ** p < 0.005, p < 0.0005 ***

**S4 Table. Quadratic regression of AIC vs. fractional power of author number.**

|  |  | **Coefficients** | | **Quad** | **Peak** | **R^2^** | | |
| --- | --- | --- | --- | --- | --- | --- | --- | --- |
| **Variable** | **Rank** | Linear | Quad | **Better** | **Power** | Linear | Quad. | Differ |
| Number | Associate | -453  *** | 554  *** | ******* | 0.409 | 0.550 | 0.995 | 0.445 |
|  | Professor | -505  *** | 469  *** | ******* | 0.538 | 0.087 | 0.9994 | 0.9907 |

Nested linear regression was used. Linear is linear coefficient, Quad is quadratic coefficient, Quad Better is the significance of the comparison between the linear and quadratic fit, Peak Power is peak fractional power and were calculated as the [(linear coefficient)/(-2*(quadratic coefficient))]. R^2^ differ is the difference between linear and quadratic regression R^2^ values, Number is number of publications and Rate is rate of publication. Associate is associate professor, and Professor is full professor. AIC values of small survival analysis model (number and rate of publication, years to hire, and moves as an assistant professor and interaction between years to hire and rate of publication) fit were plotted against fractional powers with the number and rate of publication divided by fractional powers of the author number which varied from 0.1 to 1. Significance: + p < 0.05, * p < 0.01, ** p < 0.005, and *** p < 0.0005.

S5 Table shows the mean number of years vs. estimated means using survival analysis for all professors, those working in small or large research groups and advantage of working in a larger research group.

**S5 Table. Comparison of mean years to promotion calculated using data or regression**.

| **Years to** | **Divide By** | **Research Group Size** | | | **Advantage** |
| --- | --- | --- | --- | --- | --- |
| **Promotion** | **Authors (N)** | **All** | **Small** | **Large** | **Large** |
| ASSOCIATE | N^0^ or MEAN | 6.73 | 6.78 | 6.65 | 0.13 |
|  | N^0.1^ | 6.82 | 6.88 | 6.75 | 0.13 |
|  | N^0.2^ | 6.82 | 6.88 | 6.76 | 0.12 |
|  | N^0.3^ | 6.83 | 6.88 | 6.76 | 0.12 |
|  | N^0.4^ | 6.83 | 6.87 | 6.76 | 0.11 |
|  | N^0.5^ | 6.84 | 6.87 | 6.77 | 0.10 |
|  | N^0.6^ | 6.84 | 6.87 | 6.77 | 0.10 |
|  | N^0.7^ | 6.85 | 6.87 | 6.77 | 0.10 |
|  | N^0.8^ | 6.85 | 6.87 | 6.77 | 0.10 |
|  | N^0.9^ | 6.85 | 6.88 | 6.77 | 0.11 |
|  | N^1.0^ | 6.86 | 6.88 | 6.78 | 0.10 |
| PROFESSOR | N^0^ or MEAN | 6.85 | 7.29 | 6.31 | 0.98 |
|  | N^0.1^ | 6.99 | 7.48 | 6.38 | 1.10 |
|  | N^0.2^ | 6.99 | 7.49 | 6.37 | 1.12 |
|  | N^0.3^ | 6.99 | 7.49 | 6.37 | 1.12 |
|  | N^0.4^ | 7.00 | 7.50 | 6.37 | 1.15 |
|  | N^0.5^ | 7.01 | 7.52 | 6.37 | 1.15 |
|  | N^0.6^ | 7.02 | 7.53 | 6.37 | 1.16 |
|  | N^0.7^ | 7.04 | 7.54 | 6.37 | 1.17 |
|  | N^0.8^ | 7.06 | 7.56 | 6.37 | 1.19 |
|  | N^0.9^ | 7.09 | 7.57 | 6.37 | 1.20 |
|  | N^1.0^ | 7.11 | 7.58 | 6.37 | 1.21 |

Years to promotion is the incremental years from hiring to promotion to become an associate professor or years from last promotion as estimated based on survival analysis using four variables (number and rate of publication, years to hire, and moves as an assistant professor) and one interaction term (between years to hire and rate of publication). N^0^ or MEAN is the mean of the raw data and was not estimated using survival analysis. Divide by N^k^ indicated number and rate of publication were divided by the average number of authors, N, raised to the fractional power k which varied from 0.1 to 1.0. ALL includes all professors. The mean of the number of authors (2.85 for associate professors and 3.38 for full professors) was used to separate the Small and Large research groups. Advantage Large is positive if professors working in a large research group were promoted more rapidly than those working in a small research group.

The incremental years to associate professor promotion varies little for professors working in large vs. small research groups, about 0.10 to 0.13, however for full professors there was an advantage of over a year for professors working in larger research groups. Differences between fractional powers were small, ≤ 0.3 years for associate professors and ≤ 0.22 years for full professors. There was a suggestion of a quadratic relationship, as shown in S6 Table.

**S6 Table. Quadratic regression for large group advantage vs. fractional power.**

|  |  | **Coefficients** | | **Quad** | **Peak** | **R^2^** | | |
| --- | --- | --- | --- | --- | --- | --- | --- | --- |
| **Variable** | **Rank** | Linear | Quad | **Better** | **Power** | Linear | Quad. | Differ |
|  | Associate | -0.1077  ** | 0.0720  ** | **+** | 0.74 | 0.614 | 0.865 | 0.251 |
|  | Professor | 0.1236  ** | -0.0038 | ns |  | 0.979 | 0.979 |  |

Nested linear regression was used. Quad is quadratic coefficient, Quad Better is the significance of the comparison between the linear and quadratic fit, Power is fractional power calculated as [(linear coefficient)/(-2*(quadratic coefficient))]. R^2^ Differ is the difference between linear and quadratic regression R^2^ values, Coefficients relate the advantage of working in a larger vs. small research group in years vs. fractional power which varied from 0.1 to 1 in steps of 0.1. Associate is associate professor; Professor is full professor. Small survival analysis model same as used in Table S5. Significance: + p < 0.05, * p < 0.01, ** p < 0.005, and *** p < 0.0005.

The quadratic coefficient for associate professor promotion is significant and of small magnitude; the relationship for full professors is linear with a significant, but slight positive slope. The results are striking. Professors working in larger research groups become a full professor more quickly than those working in smaller research groups, but have no advantage in years needed to gain promotion to become an associate professor. The expected fractional power relationship of 0.5 was 0.74 for associate professors and absent for full professors [9-11]. Any effect of correcting publications by dividing by different fractional powers of the number of authors, N^k^, is slight in magnitude for associate professors and small for full professors.

**References**

1. Horst P, Wallin PC, Guttman LC, Wallin FB, Clausen JA, Reed RC, et al. The prediction of personal adjustment: A survey of logical problems and research techniques, with illustrative application to problems of vocational selection, school success, marriage, and crime. New York: Social Science Research Council; 1941, p434. DOI: 10.1037/11521-000

2. Conger AJ. A Revised Definition for Suppressor Variables: A Guide to Their Identification and Interpretation. Educ Psychol Meas. 1974; 34: 35-46

3. Pandey S, Elliott W. Suppressor Variables in Social Work Research: Ways to Identify in Multiple Regression Models. J. Soc. Social Work Res. 2010; 1: 28-40.

4. Beckstead JW. Isolating and Examining Sources of Suppression and Multicollinearity in Multiple Linear Regression. Multivar. Behav. Res. 2012; 47: 224-46. DOI: [10.1080/00273171.2012.658331](http://dx.doi.org/10.1080/00273171.2012.658331)

5. Cohen J, Cohen P, West SG, Aiken LS. Applied Multiple Regression/Correlation Analysis for the Behavioral Sciences. 3rd ed. New York: Routledge; 2015.

6. Lenz G, Sahn A. Achieving Statistical Significance with Control Variables and without Transparency. Polit. Anal. 2020; 29: 356-69. DOI: <https://doi.org/10.1017/pan.2020.31>

7. Meyers LS, Gamst G, Guarino AJ. Applied Multivariate Research: Design and Interpretation. Thousand Oaks: Sage Publications; 2006.

8. Tabachnick BG, Fidel LS. Using Multivariate Statistics. 7th ed. Upper Saddle River: Pearson; 2020.

9. Bikard M, Murray F, Gans JS. Exploring Trade-offs in the Organization of Scientific Work: Collaboration and Scientific Reward. Manage. Sci. 2015; 61: 1473-95. DOI: [10.1287/mnsc.2014.2052](https://doi.org/10.1287/mnsc.2014.2052)

10. Ingram AG, Graves J, Peckham V. The Ringelmann Effect: Studies of Group Size and Group Performance. J Exp Soc Psychol. 1974; 10: 371-84.

11. Nicholls PT. Price’s square root law: Empirical validity and relation to Lakota’s law. Inf Process Manag. 1988; 24: 469-77.
